# Soy Intake and Risk of Type 2 Diabetes Among Japanese Men and Women: JACC Study

**DOI:** 10.3389/fnut.2021.813742

**Published:** 2022-01-10

**Authors:** Fangyu Yan, Ehab S. Eshak, Kokoro Shirai, Jia-Yi Dong, Isao Muraki, Akiko Tamakoshi, Hiroyasu Iso

**Affiliations:** ^1^Department of Social Medicine, Public Health, Osaka University Graduate School of Medicine, Osaka, Japan; ^2^Department of Public Health and Preventive Medicine, Faculty of Medicine, Minia University, Minia, Egypt; ^3^Advanced Clinical Epidemiology, Medical Data Science, Public Health, Graduate School of Medicine, Osaka University, Osaka, Japan; ^4^Department of Public Health, Hokkaido University Graduate School of Medicine, Sapporo, Japan

**Keywords:** soy, tofu (soybean curd), type 2 diabetes, Japan, isoflavone

## Abstract

The evidence on the protective effects of soy foods against type 2 diabetes has been inconsistent. We thought to examine the association between the dietary intakes of soy and the risk of diabetes in a prospective study encompassing 21,925 healthy Japanese men and women aged 40–79 years. A validated self-administered food frequency questionnaire determined the intakes of soy, and their associations with risk of type 2 diabetes were evaluated by the logistic regression analysis. During the 5-year follow-up period, we observed 593 new cases of type 2 diabetes (302 in men and 291 in women). There was no association between dietary intakes of soy foods and the risk of type 2 diabetes among men. Whereas among women, higher tofu intake was inversely associated with risk of type 2 diabetes; the multivariable odds ratios (ORs) of type 2 diabetes were 0.92 (95% CI: 0.69–1.21) for 3–4 times per week and 0.67 (95% CI: 0.49–0.94) for almost daily (*p*-trend = 0.03) in reference to those consuming tofu less than 3 times per week. Intakes of boiled beans and miso soup were not associated with the risk in both genders. The inverse association tended to be more evident among overweight women and postmenopaused women. In conclusion, the frequency of tofu intake was inversely associated with the risk of type 2 diabetes among women.

## Introduction

Type 2 diabetes is becoming a heavy burden worldwide. Diabetes mellitus is the 7th leading cause of death, which ignores its secondary complications, whereas it is the 3rd cause of mortality when secondary complications are also considered. According to the data published by the International Diabetes Federation (IDF) in the 9th atlas, 463 million diabetics existed worldwide in 2019. It is expected to rise by 51% and reach 578 million by 2030 and 700 million by 2045 (1). In Japan, the Ministry of Health, Labor, and Welfare reported in 2019 that 11 million people are suspected of having diabetes, with a prevalence of 19.7% in men and 10.8% in women (2).

Soy products have long been considered a source of high-quality protein and healthful fat (3). The low frequency of obesity and related metabolic disorders in Asian populations is thought to be, at least partially, the credit of high soy intake, a characteristic component in Asiatic diets (4). Several animal studies showed that a soy-based diet increased insulin sensitivity and lowered insulin requirement (5–7).

However, the evidence of the protective effects of soy foods against type 2 diabetes in humans has been inconsistent. A meta-analysis based on randomized clinical trials showed no overall significance of soy intake on improvements of fasting glucose or insulin concentrations (8). Meta-analyses based on observational studies reached inconsistent conclusions (9–12). Two meta-analyses suggested that the soy products were associated with a lower risk of type 2 diabetes mellitus (9, 10). A third meta-analysis showed that only tofu, but not total soy, was associated with the reduced risk (11). On the contrary, a fourth meta-analysis suggested a weak positive association between total legume (soy + pulses + peanuts) consumption and the risk of type 2 diabetes, while soy intake per se was not associated with the risk (12).

In the aforementioned meta-analyses, several heterogeneities related to the participants' gender and ethnicity and the type and the amount of soy food consumption under each study were discussed. Therefore, in this study, we aim to clarify the gender-specific association between the dietary intakes of three different soy foods with the risk of type 2 diabetes among the Japanese population, whose soy intake is almost 10 times higher than in western countries (13).

## Methods

### Study Population

The Japan Collaborative Cohort Study for Evaluation of Cancer Risk (JACC Study) is a large prospective cohort study sponsored by the Japanese Ministry of Education, Sports, and Science in 1988–1990. A total of 1,10,585 residents (46,395 men and 64,190 women) aged 40–79 years from 45 areas throughout Japan were enrolled in the baseline survey after having informed individual consent from the participants in 36 study areas or group consent from community leaders in 9 study areas (14). Participants completed a self-administered questionnaire at baseline, and a 5-year follow-up survey was conducted in 31 of the 45 areas involved in the baseline survey, with 46,540 participants responding. A total of 27,427 (10,589 men and 16,838 women) individuals completed the 5-year follow-up survey of diabetes and did not have a history of diabetes at baseline survey time. Of whom, 21,925 participants (8,413 men and 13,512 women) had the baseline information on soy intake, including tofu, boiled beans, and miso soup. Finally, these 21,925 participants were included in this study (Supplementary Figure S1). The study design was approved by the ethics committees of Hokkaido University and Osaka University.

### Baseline Survey

The baseline data were collected using a self-administered questionnaire that included queries on demographic characteristics, medical history, and lifestyle habits. For soy foods, the baseline questionnaire included a 40-item food frequency questionnaire (FFQ) that inquired about the diet over the past year. The frequency of tofu and boiled beans intake consisted of five levels (almost never, one or two times per month, one or two times per week, three or four times per week, and almost daily). The frequency of miso soup intake consisted of four levels (almost never, a few days per month, three or four days per week, and almost daily). The number of bowls of miso soup consumed per day was also given for those who responded to daily intake. The FFQ was validated by referring to 12-day weighed dietary records as a standard. The Spearman's correlation coefficients for the intake of soy foods between the FFQ and dietary records were 0.50, 0.30, and 0.69 for tofu, boiled beans, and miso soup, respectively (15).

### Ascertainment of Diabetes

The incidence of type 2 diabetes was defined as self-reported physician-diagnosed diabetes at the 5-year follow-up survey for participants without such a history at the baseline survey. The validity of self-reporting physician-diagnosed diabetes was evaluated by comparing self-reported diabetes with the participants' glucose concentrations or treatment history among 1,230 men and 1,837 women. Diabetes cases were defined as ≥7.8 mmol/L (≥140 mg/dL) fasting serum glucose concentration or ≥ 11.1 mmol/L (≥200 mg/dL) randomly measured concentration, or treatment with oral hypoglycemic agents or insulin (16). The sensitivity and specificity of self-reporting were 70 and 95%, respectively, for men and 75 and 98%, respectively, for women.

### Statistical Analysis

We categorized the frequency of tofu consumption into three groups: less than 3 times per week, 3 to 4 times per week, and almost daily. The frequency of miso soup consumption was categorized into four groups (less than one bowl per day, one bowl per day, two bowls per day, and at least three bowls per day). The frequency of boiled beans consumption was categorized into three groups (less than weekly, one to two times per week, and at least three times per week).

Statistical interaction was examined between the intake of soy foods groups and sex toward the risk of type 2 diabetes. Then, we calculated the sex-specific mean values and prevalence of type 2 diabetes risk factors and baseline participants' characteristics based on the categories of intake of soy foods. Using the lowest intake frequency category as a reference, we estimated the sex-specific odds ratios (ORs) and 95% confidence intervals (CIs) of having developed type 2 diabetes related to the intake of soy foods by the logistic regression analysis after adjusting for age and area of residence (Model 1). Model 2 was further adjusted for body mass index (BMI) (<18.5, 18.5–25.0, or ≥25 kg/m^2^), history of hypertension (yes or no), family history of diabetes (yes or no), sports hours (<5h/ week or ≥5h/ week), walking hours (<1h/ day or ≥1h/ day), alcohol intake (never, ex-drinker, or current drinker of 0.1–22.9, 23.0–45.9, 46.0–68.9, or ≥69.0 g ethanol/day), educational status (<18 or ≥19 years), sleep duration (<6.0, 6.0–7.0, 7.0–8.0, 8.0–9.0, or ≥9.0 h/day), smoking status (never, ex-smoker, current smoker of 1–19 cigarettes/day, or ≥20 cigarettes/day), mental stress (high or not high), having a paid job (yes or no), and nutritional factors, including intakes of total energy (quartiles), coffee (never, 1–2 cups per month, 1–2 cups per week, 3–4 cups per week, 1–2 cups per day, or ≥ 3 cups day), green tea (never, less than 2 cups per week, 3–4 cups per week, 1–2 cups per day, 3–9 cups per day, or ≥ 10 cups per day), and rice intake (1–2 bowls per day, 3 bowls per day, or ≥ 3 bowls day). These variables were associated, through previous studies, with the risk of type 2 diabetes. Mutual adjustments were made for each soy food with the other two soy foods in Model 3. Additionally, stratified analyses by the BMI (<25 and ≥ 25 kg/m^2^) among both men and women and by the menopausal status (premenopausal and postmenopausal) among the women group were conducted. The statistical significance of soy food intake interactions with BMI and menopausal status toward the risk of type 2 diabetes was also tested for crossproduct terms between each soy food intake categorical variable and dichotomous BMI and menopausal status. SAS 9.4 software (SAS Institute Inc., Cary, NC, USA) was used for all statistical analyses, and two-tailed *p*-value < 0.05 indicated statistical significance.

## Results

Table 1 shows the sex-specific mean values and prevalence of diabetes risk factor at the baseline based on the intakes of different kinds of soy foods. Men and women who had more frequent soy intake were older, more educated, and more likely to practice sports but were less likely to smoke, have hypertension, and perceived mental stress than their counterparts who consumed soy less frequently.

**Table 1:** Sex-specific baseline participants' characteristics according to intake of soy foods.

	**Tofu intake**			***p-*trend**	**Boiled beans intake**			***p*-trend**	**Miso soup intake**				***p*-trend**
	** <3 times/week**	**3–4 times/week**	**Almost daily**		**Less than weekly**	**1–2 times/week**	**≥ 3 times /week**		** <1 bowl/day**	**1 bowl/day**	**2 bowls/day**	**≥3 bowls/day**	
**Men, n**	3476	2727	2210		5202	2034	1177		2171	1531	2202	2509	
Age, year	55.9 ± 9.7	56.3 ± 9.5	57.7 ± 9.3	<0.0001	55.3 ± 9.5	57.9 ± 9.4	59.5 ± 9.0	<0.0001	56.9 ± 9.9	57.5 ± 9.7	55.9 ± 9.4	56.1 ± 9.3	<0.0001
History of hypertension, %	20.0	21.8	21.5	0.77	20.8	21.2	21.3	0.02	23.3	22.8	19.4	19.3	0.002
Current smoker, %	53.4	48.4	49.5	0.05	53.6	46.2	45.9	<0.0001	52.4	45.9	50.6	52.5	0.16
Sports≥5 h/week, %	7.1	8.4	9.2	0.10	7.0	9.0	11.5	0.005	8.8	7.5	7.5	8.4	0.01
Walking≥1 h/day, %	50.6	50.1	53.2	<0.0001	50.0	52.8	53.2	0.12	47.6	44.6	51.0	58.3	<0.0001
Higher education, %	16.6	18.9	18.4	0.06	17.1	18.3	20.3	0.01	20.5	21.4	19.7	11.7	<0.0001
High mental stress, %	22.3	22.2	20.3	0.74	23.5	20.2	17.0	0.02	25.3	22.1	22.7	17.5	<0.0001
BMI, kg/m^2^	22.5 ± 2.7	22.6 ± 2.6	22.7 ± 2.7	0.001	22.7 ± 2.7	22.4 ± 2.6	22.3 ± 2.7	0.0005	22.5 ± 2.7	22.4 ± 2.8	22.7 ± 2.6	22.7 ± 2.7	0.02
Alcohol intake, g/day	33.1 ± 22.0	33.4 ± 21.3	35.0 ± 22.1	0.0007	35.3 ± 21.9	30.9 ± 22.1	30.9 ± 20.3	<0.0001	31.9 ± 22.5	31.2 ± 20.9	33.4 ± 20.8	36.9 ± 22.4	<0.0001
Sleep duration, h	7.4 ± 1.1	7.4 ± 1.0	7.5 ± 1.1	0.0012	7.5 ± 1.1	7.4 ± 1.0	7.5 ± 1.0	0.04	7.4 ± 1.1	7.4 ± 1.0	7.4 ± 1.0	7.6 ± 1.1	<0.0001
Unemployed, %	15.8	15.8	19.1	0.61	15.4	18.1	19.6	0.002	20.9	19.8	13.4	13.7	<0.0001
≥ 1 cup of coffee/day, %	45.0	42.4	37.8	<0.0001	42.1	43.3	41.6	0.002	53.2	43.1	42.4	31.5	<0.0001
≥ 1 cup of green tea/day, %	78.2	78.3	79.4	0.64	76.7	81.2	82.1	<0.0001	79.6	79.8	79.9	75.6	0.06
≥ 3 bowls of rice/day, %	49.2	50.4	48.4	0.04	50.1	49.1	46.3	0.9	43.8	35.6	47.9	63.8	<0.0001
**Women, n**	4689	4436	4387		7942	3322	2248		4432	3331	3386	2363	
Age, year	56.7 ± 9.9	55.5 ± 9.4	56.4 ± 9.0	0.1309	55.0 ± 9.3	57.4 ± 9.5	58.6 ± 9.0	<0.0001	56.5 ± 9.9	56.3 ± 9.5	55.4 ± 9.1	56.6 ± 9.0	0.08
History of hypertension, %	21.0	19.6	21.2	0.3821	20.0	21.4	21.5	0.02	22.6	21.0	18.6	19.2	0.0002
Current smoker, %	5.1	3.8	2.8	<0.0001	4.6	3.3	2.4	<0.0001	6.0	3.4	2.8	2.5	<0.0001
Sports≥ 5 h/week, %	4.0	4.3	5.0	<0.0001	3.9	4.5	6.2	0.0003	4.4	3.8	3.7	6.4	<0.0001
Walking≥1 h/day, %	52.7	51.2	51.8	0.019	50.8	53.2	54.0	0.0007	49.5	49.8	52.7	58.2	<0.0001
Higher education, %	8.6	10.0	10.8	0.0004	9.3	10.1	10.8	<0.0001	10.8	11.1	9.5	6.1	<0.0001
High mental stress, %	20.4	19.6	20.6	0.9123	21.1	20.0	17.4	0.24	21.9	20.6	19.3	17.5	<0.0001
BMI, kg/m^2^	22.7 ± 3.1	22.8 ± 2.9	23.0 ± 3.0	0.0005	22.9 ± 3.0	22.7 ± 2.9	22.6 ± 3.0	0.0004	22.7 ± 3.0	22.7 ± 2.9	22.9 ± 2.9	23.1 ± 3.1	<0.0001
Alcohol intake, g/day	10.4 ± 14.6	8.9 ± 11.1	9.2 ± 10.6	0.0555	10.3 ± 13.2	8.2 ± 9.8	7.9 ± 10.7	0.0002	10.4 ± 13.4	8.1 ± 10.1	9.5 ± 12.4	9.7 ± 12.0	0.29
Sleep duration, h	7.1 ± 1.0	7.0 ± 1.0	7.1 ± 1.0	0.0527	7.0 ± 1.0	7.1 ± 1.0	7.1 ± 1.0	0.54	7.0 ± 1.0	7.0 ± 1.0	7.1 ± 1.0	7.3 ± 1.0	<0.0001
Unemployed, %	49.1	48.4	54.4	<0.0001	47.9	53.2	56.0	0.58	51.3	51.4	48.1	51.4	0.54
≥1 cup of coffee/day, %	43.5	44.7	37.5	<0.0001	42.3	41.8	41.0	0.002	51.9	42.4	37.0	27.6	<0.0001
≥1 cup of green tea/day, %	73.9	76.2	76.8	0.0003	73.0	79.3	79.3	<0.0001	77.1	79.0	74.0	70.1	<0.0001
≥3 bowls of rice/day, %	24.3	21.3	19.4	0.09	22.3	21.8	19.6	0.08	22.2	15.7	19.3	32.9	<0.0001

During the 5-year follow-up period, we ascertained 593 new cases of type 2 diabetes (302 in men and 291 in women). Table 2 presents the ORs (95% CIs) for type 2 diabetes according to the intake of soy foods. There was no association between dietary intakes of soy foods and risk of type 2 diabetes among men. Whereas among women, higher tofu intake was inversely associated with the risk of type 2 diabetes (*p*-trend = 0.005 in Model 1). Boiled beans and miso soup intakes were not associated with the risk in women. After controlling for type 2 diabetes traditional risk factors, the multivariable OR for type 2 diabetes among women who consuming tofu almost daily in reference to those consuming tofu <3 times per week was 0.66 (95% CI: 0.48–0.91, *p*-trend = 0.01 in Model 2), and the association was still significant after further adjustment for the intakes of other two soy foods; OR = 0.67 (95% CI:0.49–0.94, *p*-trend = 0.02 in model 3), *p*-value for sex-interaction = 0.01.

**Table 2:** Odds ratios (ORs) and 95% confidence intervals (CIs) for risk of type 2 diabetes according to intake of soy foods.

	**Tofu**			***p*-trend**	**Boiled beans**			***p*-trend**	**Miso soup**				***p*-trend**
	** <3 times/week**	**3–4 times/week**	**Almost daily**		**Less than weekly**	**1–2 times/week**	**≥3 times /week**		** <1 bowl/day**	**1 bowl/day**	**2 bowls/day**	**≥3 bowls/day**	
**Men, n**	3476	2727	2210		5202	2034	1177		2171	1531	2202	2509	
No. of Cases, n	120	95	87		195	59	48		88	59	79	76	
Age– and area–adjusted OR (95% CI)	1.00	1.06(0.80–1.40)	1.22(0.91–1.63)	0.19	1.00	0.71(0.53–0.96)	0.99(0.71–1.37)	0.40	1.00	1.06(0.75 −1.50)	1.11(0.78 −1.59)	0.96(0.65 −1.43)	0.96
Multivariable adjusted OR (95% CI)^*^	1.00	1.09(0.82–1.45)	1.26(0.93–1.7)	0.15	1.00	0.77(0.57–1.05)	1.10(0.78–1.55)	0.90	1.00	1.02(0.72–1.46)	1.09(0.76–1.58)	0.94(0.62–1.43)	0.89
Multivariable adjusted OR (95% CI)^**^	1.00	1.09(0.82–1.46)	1.25(0.91–1.7)	0.17	1.00	0.77(0.56–1.04)	1.07(0.76–1.51)	0.79	1.00	0.99(0.69–1.42)	1.07(0.74–1.55)	0.92(0.6–1.41)	0.82
**Women, n**	4689	4436	4387		7942	3322	2248		4432	3331	3386	2363	
No. of Cases, n	123	97	71		182	66	43		113	69	57	52	
Age– and area–adjusted OR (95% CI)	1.00	0.86(0.66 −1.14)	0.64(0.48 −0.87)	0.005	1.00	0.79(0.59–1.05)	0.75(0.53–1.05)	0.05	1.00	0.88(0.64 −1.21)	0.73(0.50 −1.07)	0.85(0.55 −1.32)	0.26
Multivariable adjusted OR (95% CI)^*^	1.00	0.90(0.68–1.19)	0.66(0.48–0.91)	0.01	1.00	0.82(0.61–1.10)	0.82(0.57–1.17)	0.17	1.00	0.93(0.67–1.29)	0.82(0.55–1.21)	1.00(0.63–1.59)	0.73
Multivariable adjusted OR (95% CI)^**^	1.00	0.92(0.69–1.21)	0.67(0.49–0.94)	0.02	1.00	0.82(0.61–1.10)	0.84(0.59–1.20)	0.21	1.00	1.01(0.73–1.41)	0.87(0.59–1.29)	1.05(0.66–1.67)	0.93
*P* for sex interaction	0.01				0.27				0.65				

* *Adjusted for age, area, energy, body mass index, history of hypertension, family history of diabetes, sports hours, walking hours, alcohol intake, educational status, sleep duration, smoking status, mental stress, work status, and nutritional factors (coffee, green tea, rice)*.

** *Adjusted further for intake of other two soy foods*.

There was no significant interaction with BMI (< or **≥** 25 kg/m^2^) for the associations between soy foods and risk of type 2 diabetes; *p*-interactions were > 0.05 (Table 3). However, of note, the multivariable OR (95%CI) for type 2 diabetes risk comparing the highest vs. lowest intake frequencies of tofu was 0.53 (0.31–0.91; *p*-trend = 0.03) in overweight women and 0.80 (0.53–1.23; *p*-trend = 0.31) in lean women and that for miso soup consumption in men was 3.21 (1.19–8.67; *p*-trend = 0.02) in overweight men and 0.65 (0.39–1.08; *p*-trend = 0.11) in lean men.

**Table 3:** Odds ratios (ORs) and 95% confidence intervals (CIs) for risk of type 2 diabetes according to tofu intake among different BMI groups.

	**Tofu**			***p*–trend**
	** <3 times/week**	**3–4 times/week**	**Almost daily**	
**Men, BMI <25kg/m** ^**2**^ **, n**	2770	2216	1765	
No. of cases, n	77	68	62	
Age– and area–adjusted OR (95% CI)	1.00	1.16(0.83–1.62)	1.32(0.94–1.88)	0.11
Multivariable adjusted OR (95% CI)^*^	1.00	1.17(0.83–1.65)	1.35(0.94–1.94)	0.10
Multivariable adjusted OR (95% CI)^**^	1.00	1.2(0.85–1.69)	1.42(0.98–2.05)	0.06
**Men, BMI≥25kg/m** ^**2**^ **, n**	576	438	369	
No. of cases, n	35	26	24	
Age– and area–adjusted OR (95% CI)	1.00	1.07(0.62–1.84)	1.19(0.68–2.11)	0.55
Multivariable adjusted OR (95% CI)^*^	1.00	1.29(0.71–2.35)	1.63(0.84–3.16)	0.14
Multivariable adjusted OR (95% CI)^**^	1.00	1.25(0.69–2.29)	1.47(0.75–2.88)	0.25
*P* for BMI interaction	0.91			
**Women, BMI <25kg/m** ^**2**^ **, n**	3548	3420	3333	
No. of cases, n	65	51	45	
Age– and area–adjusted OR (95% CI)	1.00	0.89(0.61–1.3)	0.82(0.56–1.22)	0.32
Multivariable adjusted OR (95% CI)^*^	1.00	0.89(0.61–1.31)	0.81(0.53–1.23)	0.32
Multivariable adjusted OR (95% CI)^**^	1.00	0.90(0.61–1.32)	0.80(0.53–1.23)	0.31
**Women, BMI≥25kg/m** ^**2**^ **, n**	918	860	913	
No. of cases, n	53	44	26	
Age– and area–adjusted OR (95% CI)	1.00	0.87(0.57–1.33)	0.48(0.29–0.79)	0.01
Multivariable adjusted OR (95% CI)^*^	1.00	0.92(0.59–1.43)	0.50(0.29–0.85)	0.01
Multivariable adjusted OR (95% CI)^**^	1.00	0.95(0.61–1.49)	0.53(0.31–0.91)	0.03
*P* for BMI interaction	0.19			

* *Adjusted for age, area, energy, body mass index, history of hypertension, family history of diabetes, sports hours, walking hours, alcohol intake, educational status, sleep duration, smoking status, mental stress, work status, and nutritional factors (coffee, green tea, rice)*.

** *Adjusted further for intake of other two soy foods (boiled beans and miso soup)*.

Similarly, no significant interaction by the women's menopausal status was found for the inverse association between tofu intake and risk of type 2 diabetes (*p*-interaction = 0.34) (Table 4). The multivariable OR (95% CI) that compares the almost daily tofu intake to <3 times/week was 0.70 (0.36–1.40; *p*-trend = 0.30) in premenopausal women and 0.64 (0.43–0.93; *p*-trend = 0.02) in postmenopausal women.

**Table 4:** Odds ratios (ORs) and 95% confidence intervals (CIs) for risk of type 2 diabetes according to tofu intake among premenopausal and postmenopausal women.

	**Tofu**			***p*-trend**
	** <3 times/week**	**3–4 times/week**	**Almost daily**	
**Premenopausal women, n**	1553	1536	1310	
No. of cases, n	29	22	19	
Age– and area–adjusted OR (95% CI)	1.00	0.77(0.44–1.36)	0.80(0.44–1.48)	0.45
Multivariable adjusted OR (95% CI)^*^	1.00	0.78(0.43–1.43)	0.68(0.34–1.33)	0.24
Multivariable adjusted OR (95% CI)^**^	1.00	0.80(0.43–1.47)	0.70(0.36–1.40)	0.30
**Postmenopausal women, n**	3136	2900	3077	
No. of cases, n	94	75	52	
Age– and area–adjusted OR (95% CI)	1.00	0.88(0.64 −1.20)	0.59(0.42 −0.84)	0.004
Multivariable adjusted OR (95% CI)^*^	1.00	0.92(0.67–1.27)	0.63(0.44–0.92)	0.02
Multivariable adjusted OR (95% CI)^**^	1.00	0.94(0.68–1.30)	0.64(0.43–0.93)	0.02
*P* for menopausal status interaction	0.34			

* *Adjusted for age, area, energy, body mass index, history of hypertension, family history of diabetes, sports hours, walking hours, alcohol intake, educational status, sleep duration, smoking status, mental stress, work status, and nutritional factors (coffee, green tea, rice)*.

** *Adjusted further for intake of other two soy foods (boiled beans and miso soup)*.

## Discussion

In this prospective population-based cohort study of Japanese men and women, we found that among the soy foods (tofu, boiled beans, and miso soup), tofu intake was associated with the reduced risk of type 2 diabetes among women but not among men. The association tended to be more pronounced among overweight and postmenopausal women than lean and premenopausal women; however, no significant effect modifications were detected.

Previous epidemiological studies reported inconsistent associations between intake of soy foods and the risk of diabetes (17–21). The discrepant findings could be attributed to differences in the study design, ethnicity of the studied population, and type and assessment of intake of soy foods. For the evidence in Japan, our findings are consistent with that of the Takayama cohort study of 13,521 (5,883 men and 7,638 women) residents aged 35–69 years and followed for a median 10-year period (17). For that study, the multivariable HR (95% CI) or type 2 diabetes risk for the highest vs. lowest tertiles of total soy intake was 0.45 (0.30–0.68; *p*-trend < 0.001) among women and 1.02 (0.74–1.42; *p*-trend = 0.94) among men, but the analyses for specific soy foods were not provided (17). On the contrary, the findings from 25,872 men and 33,919 women aged 45–75 years in the Japan Public Health Center-based Prospective (JPHC) study indicated no association between intake of total soy products and the risk of 5-year incidence of type 2 diabetes in either gender, and again, the analyses for specific soy foods were not provided (18).

The evidence outside Japan was also inconsistent. Matching our findings, a Vietnamese case-control study of 599 patients with type 2 diabetes and 599 age- and sex-matched controls showed that the multivariable OR (95 % CI) of type 2 diabetes for the highest vs. lowest quintiles of intake was 0.66 (0.42–1.02; *p*-trend = 0.009) for fresh tofu, 0.51 (0.35–0.75; *p*-trend = 0.001) for fried tofu, and 0.31 (0.21–0.46; *p*-trend < 0.001) for total soy foods (19). However, a pooled analysis of 3 large cohorts of US populations (63,115 women from the nurses' health study, 79,061 women from the nurses' health study II, and 21,281 men from the health professionals follow-up study) showed no association between consumption of soy foods with the risk of type 2 diabetes; the multivariable HR (95% CI) for ≥1 serving/week compared with no consumption was 0.93 (0.83–1.03; *p*-trend = 0.14) (20). On the contrary to our finding, the Multiethnic Cohort (MEC) study showed a positive association between soy intake and the risk of type 2 diabetes among 29,719 Caucasians, HR (95% CI) for consuming ≥ 10 g/d vs. <5 g/d was 1.22 (1.02–1.46) in men and 1.44 (1.16–1.80) in women, respectively; among 35,141 Japanese Americans, HR (95% CI) was 1.14 (1.01–1.28) in men and 1.11 (0.98–1.26) in women, respectively; and among 10,484 Native Hawaiians; HR (95% CI) was 1.29 (1.08–1.53) in men and 1.19 (1.02–1.40) in women (21).

The conclusions of the available meta-analyses also showed the same inconsistent result. According to a meta-analysis of 24 randomized trials with a total of 1,518 subjects, there was no overall impact of soy intake on improvements of fasting glucose [mean (95% CI) difference was 20.69 mg/dL (21.65, 0.27 mg/dL), *p* = 0.16] or insulin concentrations [20.18 mg/dL (20.70, 0.34 mg/dL), *p* = 0.50] (8). On the contrary, a meta-analysis of 19 independent reports from 8 observational studies (6 cohort and 2 cross-sectional) with a total of 1,74,561 participants concluded that the consumption of soy foods was associated with a lower risk of type 2 diabetes mellitus. The overall relative risk (RR) (95% CI) was 0.77 (0.66–0.91) and the association was evident in women, RR = 0.65 (0.49–0.91) but not in men, RR = 0.82 (0.58–1.16), and in Asian population, RR = 0.73 (0.61–0.88) but not in non-Asians, RR = 1.05 (0.88–1.25) (10). In a recent meta-analysis of 15 prospective studies involving 11,232 cases among 2,71,709 individuals among the soy foods, only tofu was inversely associated with the risk of type 2 diabetes; RR (95% CI) was 0.92 (0.84, 0.99) for tofu, 0.89 (0.71–1.11) for soy milk, and 0.83 (0.68–1.01) for total soy intake (11).

Although we did not find significant interactions by BMI on the associations between soy foods and risk of type 2 diabetes, the inverse association of tofu was more evident in overweight women. The evidence on effect modifications by BMI from previous studies was inconclusive. Soy intake was associated with the reduced risk of type 2 diabetes in overweight but not lean men of the Saku study (22) and women of the JPHC study (18). However, the Takayama study reported the reduced risk with soy intake in lean but not overweight women (17). On the contrary, the weak positive association between soy intake and the risk of diabetes was confined to overweight and obese men and women of Caucasians, Hawaiians, and Japanese Americans in the MEC study (21). On the other hand, no effect modifications by BMI were found in the Vietnamese case-control study (19), the Shanghai Women's Health study (23), the Singapore Chinese Health Study (24), and the USA cohorts (20).

We do not have a clear explanation for the discrepant directions of BMI effect modification on the association between soy intake and the risk of type 2 diabetes. However, according to BMI cut point and also ethnicity, the amount of soy consumption and the type of soy foods varied between those previous studies. The MEC study (21) mentioned that the mean soy intake among Japanese American entity was 14.5 g, which was much lower than the 88 g among JPHC study (18) population and 87.1 g among men and 81.7 g among women in Takayama study (17). The authors of the MEC study assumed that these different soy intakes might affect the BMI interaction. The low levels of soy intake would not lead to the effect modification by BMI in the association between soy intake and the risk of type 2 diabetes. Alternatively, the differences may also be due to a random variation, residual confounding, or other dietary or lifestyle factors correlated with soy food consumption, BMI, and risk of type 2 diabetes. We recommend further longitudinal and clinical trials to assess the effect modification by BMI.

The significant inverse association between tofu intake and the risk of type 2 diabetes in postmenopausal women in our study matches the finding of the JPHC study where a tendency toward reduced risk was observed in the fourth quintile of total soy intake in postmenopausal women: HR (95% CI) = 0.69 (0.48–1.00; *p*-trend = 0.49) and premenopausal women with HR (95% CI) = 0.79 (0.57–1.09; *p*-trend = 0.66), *p-*interaction = 0.36 (18). However, the interaction by the menopausal status was not statistically significant in our study, in the JPHC study and other studies (17, 18, 20, 23, 24).

There are several potential mechanisms for soy foods' protective function against type 2 diabetes. Soy-derived protein and isoflavone contents could be the key protective factors (10). The Chinese Singaporean study (24) indicated that the isoflavone intake tended to be associated with a lower risk of diabetes; HR= 0.76 (0.58–1.00; *p*-trend = 0.08) for the highest vs. lowest quintiles of isoflavone intake. The previously mentioned Vietnamese case-control study showed that the multivariable ORs (95% CIs) for type 2 diabetes were 0.35 (0.24–0.49 *p*-trend < 0.001) for the highest (>23.2 mg/day) vs. lowest (≤ 12.6 mg/day) tertiles of major isoflavones intake, 0.36 (0.24–0.49; *p*-trend < 0.001) for daidzein, 0.35 (0.26–0.50; *p*-trend < 0.001) for genistein, and 0.37 (0.26–0.53; *p*-trend < 0.001) for glycitein (19). Total isoflavones were inversely associated with the risk of type 2 diabetes in the pooled analysis of the 3 US cohorts; the HR in the highest quintile of isoflavones vs. the lowest was 0.89 (0.83–0.96; *p*-trend = 0.009) (20). Isoflavones in the soy may inhibit insulin release from the pancreas and act as α-glucosidase inhibitors that restrict the intestinal brush border uptake of glucose (25). Also, isoflavone genistein could increase the beta-cell proliferation by ERK1/2 (26) and cAMP/PKA(27) pathways and enhance cell replication (28, 29). Moreover, pharmacological actions of isoflavones on glycemic control include a tyrosine kinase inhibitory action, changes in insulin receptor numbers and affinity, intracellular phosphorylation, and alterations in glucose transport (25, 30, 31). In addition to the isoflavones, soy protein may reduce insulin resistance by activating peroxisome proliferator-activated receptors (PPARs), which are nuclear transcription factors that regulate the expression of genes involved in glucose homeostasis, lipid metabolism, and fatty acid oxidation (32–34).

As for strengths, this study was based on a large sample of the community-based population, used a validated FFQ, and controlled for a wide range of potential confounding factors. As for limitations, the outcome variable was based on self-report; however, as shown above, there were high sex-specific sensitivity and specificity for the self-reported physician-diagnosed diabetes in our participants (35). Second, the data were collected from 36% of the eligible participants because the 5-year follow-up survey was limited to some but not to all the baseline study areas. However, no significant differences in participants' characteristics such as age, BMI, and other variables were found between individuals who responded or did not respond to the 5-year survey (36). Third, the dietary questionnaire did not include the data on portion size or some soy foods such as natto and soy milk; therefore, it was not possible to estimate the intake of total soy isoflavones. Finally, the residual confounding, such as by the glycemic index or load, cannot be eliminated.

In conclusion, soy intake from tofu was inversely associated with the risk of diabetes among women but not among men. The inverse association tended to be more evident among overweight and postmenopausal women.

## Data Availability Statement

The raw data supporting the conclusions of this article will be made available by the authors, upon justified requests to the steering committee of the JACC study.

## Ethics Statement

The JACC study protocol was approved by the Ethics Committees of Hokkaido University, Nagoya University, and Osaka University. The patients/participants provided their written informed consent to participate in this study.

## Author Contributions

FY, EE, and HI designed the study and methods of the analyses. FY drafted the manuscript. EE, KS, J-YD, IM, AT, and HI provided a critical review of the content. FY is the first author and HI is the corresponding author who both have the primary responsibility of the content. All authors contributed to the revisions and read and approved the final manuscript.

## Funding

The JACC study was supported by grants-in-aid for Scientific Research from the Ministry of Education, Culture, Sports, Science, and Technology of Japan (MEXT) (Monbusho); grants-in-aid for Scientific Research on Priority Areas of Cancer; and grants-in-aid for Scientific Research on Priority Areas of Cancer Epidemiology from MEXT (MonbuKagaku-sho) (Numbers 61010076, 62010074, 63010074, 1010068, 2151065, 3151064, 4151063, 5151069, 6279102, 11181101, 17015022, 18014011, 20014026, 20390156, and 26293138); grants-in-aid from the Ministry of Health, Labor and Welfare, Health and Labor Sciences research grants, Japan (Research on Health Services: H17–Kenkou−007; Comprehensive Research on Cardiovascular Disease and Life–Related Disease: H18–Junkankitou [Seishuu]–Ippan−012; H19–Junkankitou [Seishuu]–Ippan−012; H20–Junkankitou [Seishuu]–Ippan−013; H23–Junkankitou [Seishuu]–Ippan−005; H26-Junkankitou [Seisaku]-Ippan-001; H29–Junkankitou–Ippan−003; 20FA1002); National Cancer Center Research and Development Fund (27-A-4, 30-A-15, 2021-A-16) and JSPS KAKENHI Grant Numbers JP 16H06277 and JP25330039.

## Conflict of Interest

The authors declare that the research was conducted in the absence of any commercial or financial relationships that could be construed as a potential conflict of interest.

## Publisher's Note

All claims expressed in this article are solely those of the authors and do not necessarily represent those of their affiliated organizations, or those of the publisher, the editors and the reviewers. Any product that may be evaluated in this article, or claim that may be made by its manufacturer, is not guaranteed or endorsed by the publisher.
