# Supplementary figures and images for: Soy Intake and Risk of Type 2 Diabetes Among Japanese Men and Women: JACC Study
Source: Front Nutr. 2022 Jan 10;8:813742. doi: 10.3389/fnut.2021.813742 (PMC8784605; doi:10.3389/fnut.2021.813742)

Supplemental Figure 1. Participants' flow chart

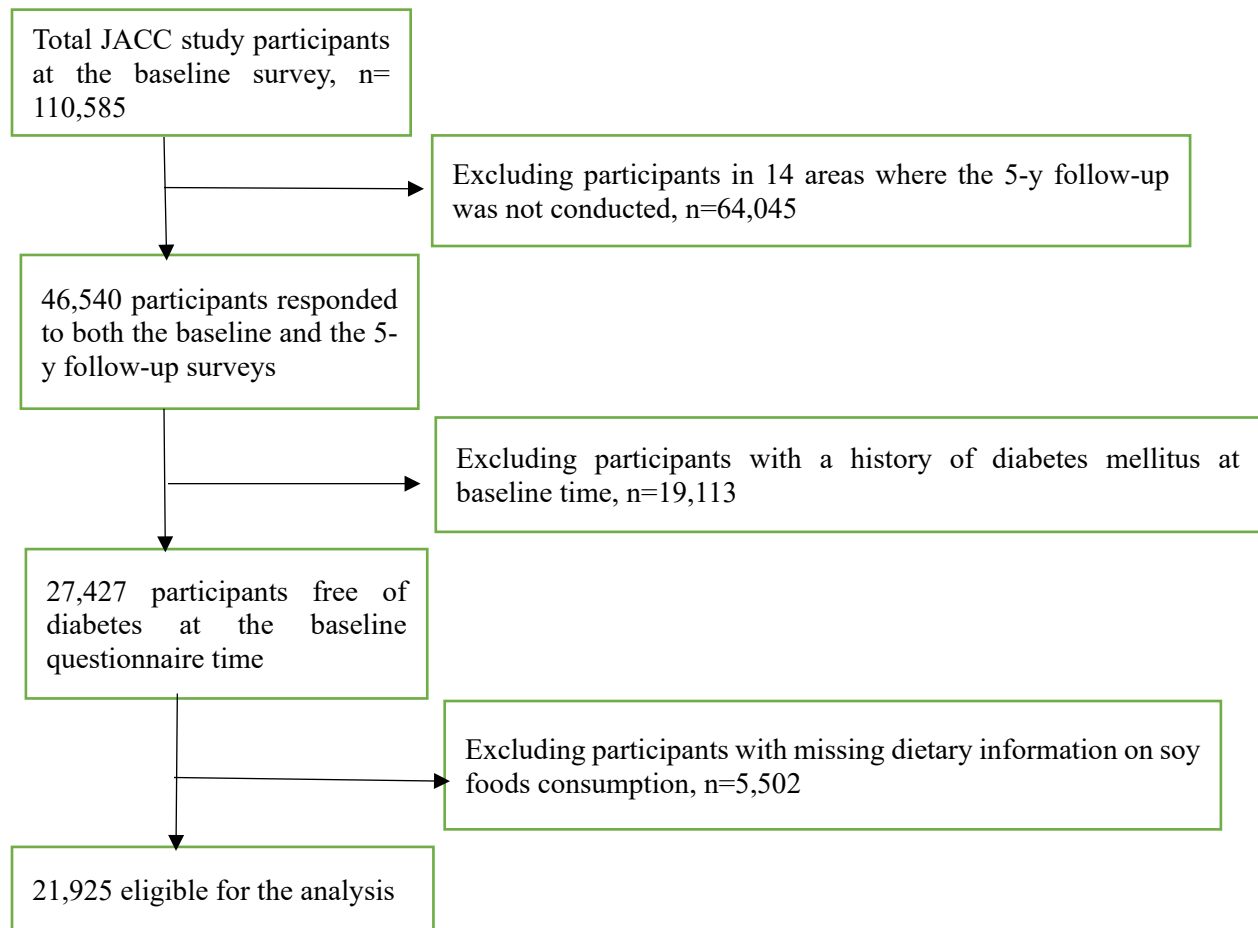

Supplement: Supplementary file 1 [file Image_1.pdf]
